# Structure of Rhomboid Protease in Complex with β-Lactam Inhibitors Defines the S2′ Cavity

**DOI:** 10.1016/j.str.2013.03.013

**Published:** 2013-06-04

**Authors:** Kutti R. Vinothkumar, Olivier A. Pierrat, Jonathan M. Large, Matthew Freeman

**Affiliations:** 1 MRC Laboratory of Molecular Biology, Francis Crick Avenue, Cambridge CB2 0QH, UK; 2 Centre for Therapeutics Discovery, MRC Technology, 1-3 Burtonhole Lane, Mill Hill, London NW7 1AD, UK

## Abstract

Rhomboids are evolutionarily conserved serine proteases that cleave transmembrane proteins within the membrane. The increasing number of known rhomboid functions in prokaryotes and eukaryotes makes them attractive drug targets. Here, we describe structures of the *Escherichia coli* rhomboid GlpG in complex with β-lactam inhibitors. The inhibitors form a single bond to the catalytic serine and the carbonyl oxygen of the inhibitor faces away from the oxyanion hole. The hydrophobic N-substituent of β-lactam inhibitors points into a cavity within the enzyme, providing a structural explanation for the specificity of β-lactams on rhomboid proteases. This same cavity probably represents the S2′ substrate binding site of GlpG. We suggest that the structural changes in β-lactam inhibitor binding reflect the state of the enzyme at an initial stage of substrate binding to the active site. The structural insights from these enzyme-inhibitor complexes provide a starting point for structure-based design for rhomboid inhibitors.

## Highlights

• Structures of *E. coli* GlpG, in complex with three different β-lactam inhibitors
• The acyl enzyme structures define the S2′ substrate binding site in GlpG
• Biochemical analysis reveals a preference for large hydrophobic groups at S2′ cavity
The GlpG-β-lactam structures reveal the changes essential for formation of S2′ cavity

## Graphical Abstract

**Figure:**
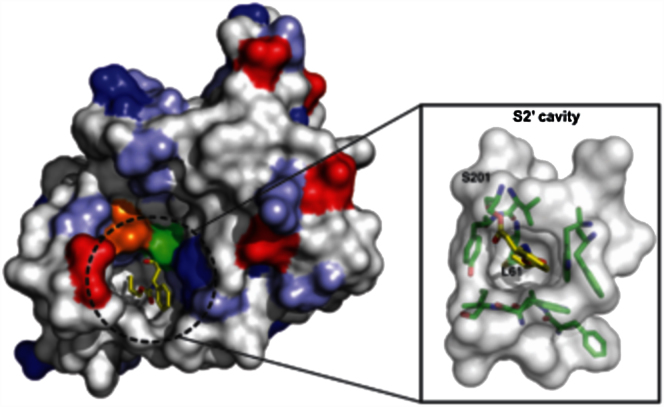

## Teaser

The structures of the *E. coli* rhomboid GlpG, an intramembrane serine protease in complex with β-lactam inhibitors, reported by Vinothkumar et al., reveal the nature and formation of the S2′ cavity. The differences in this cavity may form the basis of substrate/inhibitor specificity and structure-based drug design.

## Introduction

Recent identification of intramembrane proteases has revealed a new strategy for cellular regulation, whereby membrane proteins inactive in their membrane-bound form are activated by site-specific intramembrane proteolytic cleavage within the transmembrane (TM) helix. The domains that are released upon proteolysis move to new locations to carry out specific biologic functions (Brown et al., 2000). Rhomboids are a family of intramembrane proteases that use a catalytic dyad of serine and histidine for proteolysis of TM substrates (Freeman, 2008). Beyond their initial identification in *Drosophila* as primary regulators of the EGF receptor signaling pathway (Wasserman et al., 2000), the expanding biologic roles of rhomboids include protein translocation, parasite invasion, and mitochondrial remodeling (Freeman, 2008; Urban and Dickey, 2011). Understanding how rhomboids cleave their substrate TM domains is an active area of research, and the importance of rhomboids in various signaling pathways has highlighted them as attractive drug targets.

The structures of prokaryotic homologs of rhomboid proteases provided the first views of the architecture of an intramembrane protease family, revealing the active site in a water-filled environment surrounded by TM helices (Ben-Shem et al., 2007; Lemieux et al., 2007; Wang et al., 2006; Wu et al., 2006). Substrates are thought to interact with the enzyme through a gap between TM helices. Although rhomboids do not recognize a simple primary sequence in their substrate, a widespread substrate motif has been identified. This motif reveals that for many rhomboids, there is a preference for amino acids with small side chains at the P1 position, where the peptide bond cleavage occurs. On either side of this scissile bond, hydrophobic residues might play a role in increasing the specificity of the substrate (Moin and Urban, 2012; Strisovsky et al., 2009). It was noted very early that, besides isocoumarins, many of the classical serine protease inhibitors were ineffective against rhomboids, thus raising the question of whether rhomboids use a distinct mechanism for catalysis (Urban et al., 2001; Urban and Wolfe, 2005). However, fluorophosphonates have recently also been shown to inhibit rhomboids (Sherratt et al., 2012; Xue and Ha, 2012). The structures of rhomboid proteases in complex with isocoumarins and fluorophosphonates have extended our understanding of how a substrate might bind at the active site, what associated structural changes in the enzyme might occur, and a plausible mechanism for intramembrane proteolysis (Vinothkumar et al., 2010; Xue et al., 2012; Xue and Ha, 2012).

Recently, monocyclic β-lactams (also called monobactams) were identified as inhibitors of rhomboid proteases (Pierrat et al., 2011). Using chymotrypsin as a control serine protease, the parent β-lactam was used to design inhibitors with improved selectivity and potency for rhomboid proteases. These inhibitors were also shown to have some activity in vivo, both in *Escherichia coli* and in mammalian cells. The structure-activity relationship (SAR) of the inhibitors highlighted key chemical groups that were essential for activity and potency against rhomboid proteases (Pierrat et al., 2011).

To address the mechanism of β-lactam inhibition and its mode of binding to rhomboids, we determined structures of *E. coli* GlpG in complex with three different β-lactam inhibitors. The acyl enzyme structures define the S2′ substrate binding site in GlpG and reveal a preference for large hydrophobic groups in this position. Comparisons with previously published rhomboid structures highlight the changes essential for initial binding of the substrates and formation of the S2′ cavity. The differences in the nature of residues lining the S2′ cavity in rhomboids could form the basis for observed selectivity and specificity of β-lactams and substrates.

## Results

### Structures of GlpG in Complex with β-Lactams

**Figure 1:**
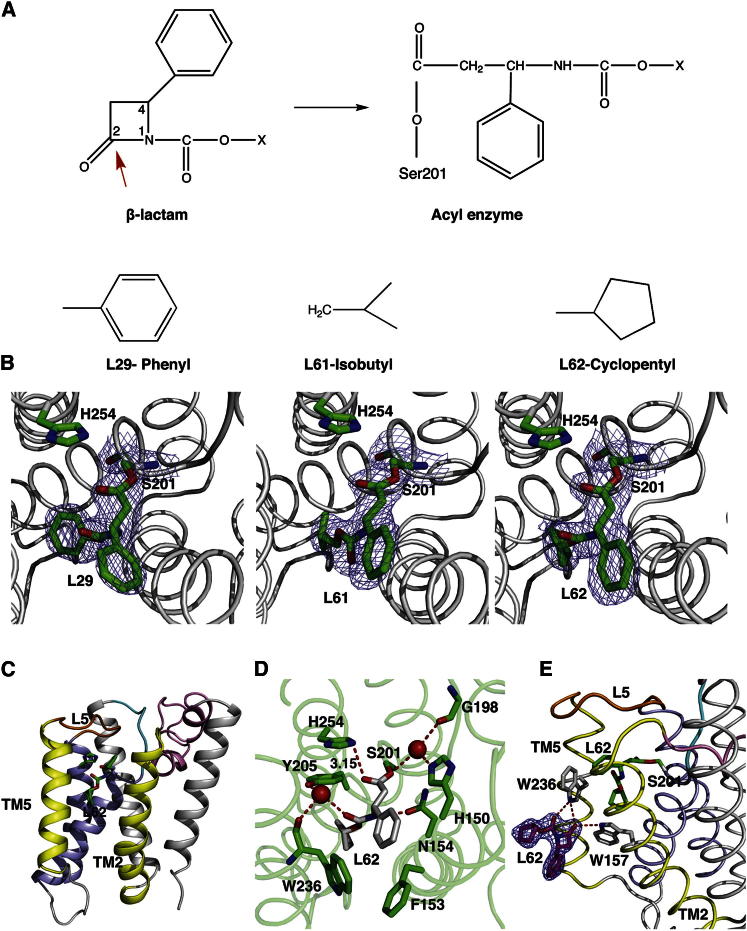
Structures of GlpG in Complex with β-Lactam Inhibitors (A) Mechanism of β-lactam inhibition of serine proteases. The nucleophilic attack of the carbonyl group at position 2 of the β-lactam ring by catalytic serine results in ring opening to form an acyl enzyme (Powers et al., 2002). (B) The different N-substituent in each of the structures is described here. A 2Fo-Fc map (blue mesh) contoured at 1.0 σ around the catalytic serine, and the inhibitors are shown in stick representation. (C) An overview of the GlpG in complex with L62. The phenyl group at position 4 points toward the gap between TM2 and TM5 (yellow). The substituent at the carbamate points into the enzyme. The active site residues, S201 and H254 and the inhibitor, are shown in stick representation. (D) Interactions of L62 with GlpG. Most interactions between the inhibitors and the enzyme are common in all three structures; the interaction of L62 is shown here. Water molecules are shown as red spheres and the hydrogen bonds are shown as red lines. The carbonyl oxygen points away from the oxyanion hole and is close to the side chain of H254. A water molecule forms hydrogen bonds with the carbamate oxygen of L62, the hydroxyl of Y205, and the backbone carbonyl of W236. Residues H150, G198, and S201 hydrogen-bond with another water molecule, which in other inhibitor structures of GlpG is displaced. The hydrogen bond between the nitrogen atom of the inhibitor and the side chain of N154 further strengthens the interaction between the enzyme and inhibitor. (E) A 2fo-fc map at 1σ (blue mesh) drawn around the uncleaved β-lactam inhibitor (magenta) at the TM2/TM5 interface. The side chains of amino acids (carbon atoms shown in gray) that hydrogen-bond (red lines) with the oxygen atoms of this external inhibitor include W236 and W157. Note that the C4 carbon of the uncleaved L62 has the opposite chirality of the L62 covalently bound to S201 (shown in green stick representation). See also Figures S1 and S2.

The inhibition of serine proteases by β-lactams involves the nucleophilic attack by the serine hydroxyl group on the carbonyl group of the inhibitor, resulting in opening of the β-lactam ring (Powers et al., 2002) (Figure 1A). Initial maps after molecular replacement in many data sets of GlpG crystals soaked with the inhibitors show the presence of continuous density at the active serine, indicating the formation of an ester bond between the enzyme and inhibitors (Figure 1B). All the structures described here derive from the same parent compound with a carbamate attached to the nitrogen atom (Pierrat et al., 2011). The carbamate substituent is a phenyl (inhibitor L29), isobutyl (inhibitor L61), or a cyclopentyl (inhibitor L62). The crystals of GlpG soaked with all β-lactams described here diffracted to 2.2–2.4 Å resolutions (Table 1) and are very similar, with minor differences in the loop regions (Figure 1C; Figure S1 and Table S1 available online). A complete loop5 (residues 245–249), with the exception of F245 side chain, could be modeled into the L62 structure. While in the L61 structure, all residues of loop5 except for F245 could be modeled. We have included two data sets of GlpG soaked with L29, which are similar but differ in map quality in certain regions of protein and water molecules (Figure S1; Table S1). In the first data set, which diffracts to 2.2 Å, loop5 is disordered, while in the second data set, which diffracts to 2.4 Å, the main chain atoms for residues 245–247 of loop5 could be modeled. Although a racemic mixture was used for soaking, the best fit to the density was observed for the R-enantiomer. The phenyl ring at position 4 of the β-lactams (Figure 1A), which is common to all three inhibitors, points into the gap between TM2 and TM5 toward the putative bilayer. The carbamate substituents point into the interior of the enzyme (Figures 1C and 1D).

**Table 1:** Data Collection and Refinement Statistics

Parameter	L29 Data Set 1 (3ZMI)	L29 Data Set 2 (3ZOT)	L61 (3ZMJ)	L62 (3ZMH)
**Cell Dimensions**				

a, b, c (Å)	110.5, 110.5, 128.5	110.2, 110.2, 128.9	110.2, 110.2, 128.2	110.1, 110.1, 128.5
γ (°)	120	120	120	120
Resolution (Å)^a^	55.2–2.2 (2.3–2.2)	53.4–2.4 (2.49–2.4)	53.2–2.3 (2.4–2.3)	76.6–2.30 (2.4-2.3)
*R*_merge_	0.062 (0.451)	0.072 (0.457)	0.068 (0.589)	0.113 (0.678)
*I*/σ*I*	16.2 (3.8)	12.6 (3.1)	14.4 (3.0)	10.9 (2.6)
Completeness (%)	99.3 (100)	99.5 (99.8)	99.2 (99.9)	100 (99.9)
Redundancy	5.2 (5.3)	4.8 (5.0)	5.2 (5.2)	5.9 (6.1)
Wilson B factor (Å^2^)	43.2	37.2	40.9	34.2

**Refinement**				

Resolution (Å)	44.8–2.2	53.4–2.4	53.2–2.3	76.6–2.3
No. of reflections	15,423	11,900	13,416	13,542
*R*_work_ / *R*_free_^b^	0.217/0.261	0.198/0.252	0.197/0.231	0.188/0.23

**No. of Atoms**				

Total	1,482	1,540	1,513	1,564
Protein	1,393	1,421	1,431	1,445
Ligand^c^	20	20	18	19
Heteroatoms^c^	39	70	37	62
Water	30	29	27	38

**B-Factors (Å**^**2**^**)**				

Total	46.6	38.9	42.0	34.7
Protein	46.1	38.2	41.6	34.2
Ligand	56.2	46.6	53.7	36.3
Heteroatoms	58.0	51.0	50.6	44.9
Water	47.5	37.6	42.1	37.1

**Rmsd**				

Bond lengths (Å)	0.008	0.008	0.007	0.007
Bond angles (°)	1.05	1.1	1.01	1.02

Rmsd, root-mean-square deviation.

a Values in parentheses are for the highest-resolution shell.

b A subset of reflections (5%) imported from apoenzyme (2XOV) was used for calculation of R_free_ and remaining (95%) reflections was used for calculation of R_work_.

c Heteroatoms denote detergent, ions, and exogenous β-lactam. Ligand denotes the β-lactam covalently bonded to S201.

A number of polar and hydrophobic interactions between the inhibitor and amino acid residues in the enzyme are observed. The carbonyl oxygen of the inhibitors points away from the oxyanion hole but is close to the Nε of H254 and the observed distance varies between 3.15 and 3.5 Å (Figure 1D; Figure S1). Because the carbonyl oxygen points away from the oxyanion hole, this space is occupied by a water molecule as in the apoenzyme and hydrogen-bonds to the side chains of H150, S201, and the backbone of G198. The interaction of inhibitor with the enzyme is further stabilized by a hydrogen bond between the nitrogen atom of the inhibitor and the side chain of N154. In the L29 and L62 structures, the carbamate oxygen of the inhibitor hydrogen-bonds to a water molecule, which in turn hydrogen-bonds to the side chain hydroxyl of Y205 and backbone carbonyl of W236. This interaction is absent in the L61 structure because the carbamate oxygen points toward TM5 (Figure S1F). The phenyl group at position 4 interacts with hydrophobic residues including M149, F153, W157 from TM2, W236 from TM5, and M247 from loop5 and has rotational freedom. In the L29 structure, the aromatic ring is rotated ∼90° when compared to the L61 and L62 structures (Figure 1B; Figure S1).

In the structure of GlpG in complex with L62, an additional density was observed at the interface between TM2 and TM5. The shape of the density suggested that it might represent a second inhibitor molecule, which is consistent with the high concentrations of inhibitor used in the soak. The best fit was observed for an uncleaved L62 molecule with an intact β-lactam ring (Figure 1E). The modeled inhibitor fits nicely into a groove formed between TM2 and TM5 (Figure S2). The side chains of W157 and W236 form a hydrogen bond with the oxygen atoms of the inhibitor and hydrophobic interactions between the β-lactam and residues of TM2 and TM5, in particular F153, W157, F232 and W236, are observed.

### S2′ Cavity

**Figure 2:**
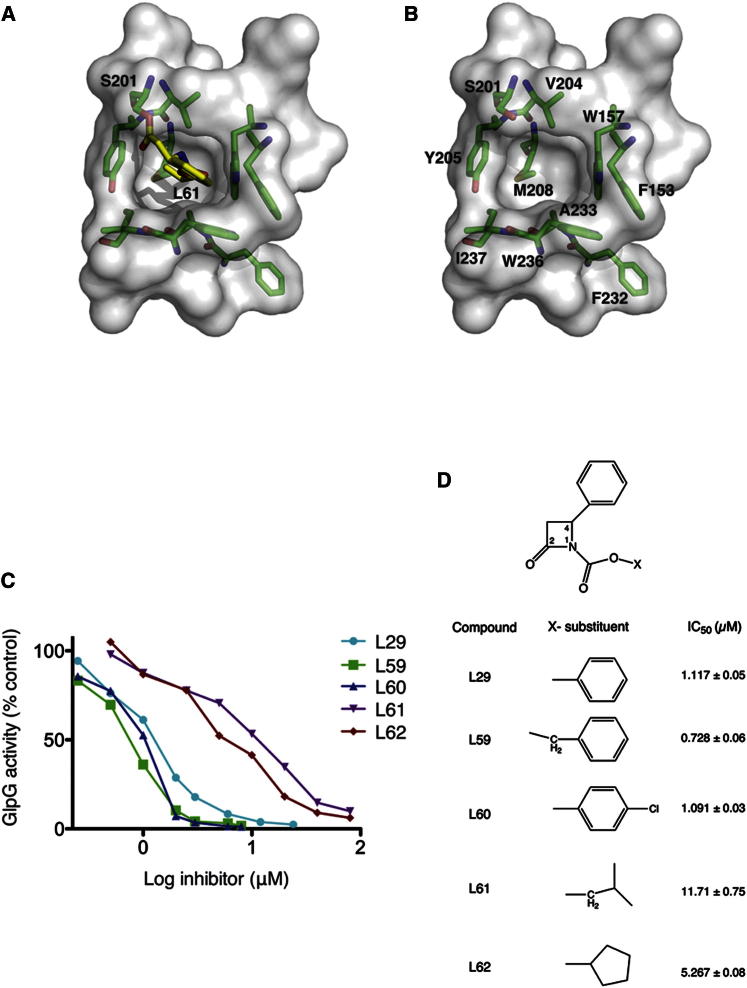
S2′ Cavity of GlpG (A) In all the structures described here, the substituent at the carbamate position points into a cavity in GlpG, formed only upon inhibitor binding in this crystal form. In this example, inhibitor L61 is shown pointing into the cavity. (B) This cavity, hypothesized as the S2′ substrate binding site, is largely hydrophobic formed by residues from TM2, TM4, and TM5. The side chain of M208 forms the base of the cavity. The aromatic rings of W157, Y205, and W236 form the walls of the cavity. The side chains of V204, A233, and I237 point into the cavity. (C) A typical plot used to estimate the IC_50_ values of inhibitors. After inhibition of GlpG (350 nM) with different concentrations of inhibitors, substrate (3.5 μM) was added and incubated for 45 min at 37°C. The cleaved products were separated by SDS gels. The intensity of the bands were quantified with ImageJ and the activity (as a percentage of control) was plotted against the inhibitor concentration. The data were fit to a four-parameter dose-response curve for determination of the IC_50_ values. (D) A table showing the average IC_50_ values (± SE) of different substituents of the inhibitor from three independent experiments. Compounds bearing larger hydrophobic groups are more potent inhibitors of GlpG. See also Figure S3.

Based on the previously published isocoumarin structure, we predicted that upon inhibitor binding, a hydrophobic cavity is formed downstream of the active site, which could represent the S2′ substrate binding site of GlpG (where the P2′ residue of substrate interacts) (Vinothkumar et al., 2010). In all the structures described here, this cavity is filled with hydrophobic carbamate substituents (Figure 2A). Residues from TM 2, TM 4, and TM 5 form the cavity. The side chain of M208 forms the base of the cavity, while the aromatic rings of W157, Y205, and W236 form the sides of the wall. Residues V204 in TM4, and A233 and I237 in TM5 also form part of the cavity (Figure 2B). To address a possible preference for certain chemical motifs binding in the S2′ cavity, we analyzed the influence of different hydrophobic carbamate groups on GlpG inhibition, which revealed an interesting correlation between size and potency (Figures 2C and 2D; Figure S3). The larger hydrophobic groups such as phenyl (L29), benzyl (L59), or 4-chlorophenyl (L60) inhibited GlpG more potently. In contrast, introduction of smaller and less hydrophobic groups such as a cyclopentane ring or isobutyl group showed a higher half maximal inhibitory concentration (IC_50_) value (Figure 2D). It is evident that the best fit for the S2′ cavity is achieved by larger hydrophobic groups such as an aryl ring (L29), explaining why the smaller isobutyl group is less active.

The formation of a single covalent bond between β-lactam inhibitors and the enzyme indicates that it should be possible to regenerate the enzyme activity by hydrolysis of the ester bond. Indeed, upon rapid dilution of inhibitor-bound rhomboid protease, a modest recovery of the activity was observed (Pierrat et al., 2011). To ascertain whether different hydrophobic groups affect the rate of reactivation of GlpG, the spontaneous recovery of activity of the β-lactam-inhibited enzyme over time was monitored (Figure S4A). Slow recovery of activity was observed for all inhibitors. GlpG inhibited with β-lactams L29 or L60 showed 50% recovery of wild-type (WT) activity within 6 and 3.5 hr, respectively. β-lactams with smaller hydrophobic groups, L61 and L62, required >24 hr to attain 50% WT activity (Figures S4A and S4B). The benzyl carbamate (L59) showed an intermediate behavior with 50% activity regained after ∼17 hr. This process of reactivation could be accelerated by the addition of hydroxylamine, a nucleophile more powerful than water. Incubation of enzyme-inhibitor complex with hydroxylamine restored 70%–80% activity of GlpG within 30 min (Figure S4C). Analogous to chymotrypsin, where the rate of deacylation was observed to be dependent on the chemical groups (Ingles et al., 1966), the difference in the rate of deacylation of β-lactams in GlpG may reflect the nature of each hydrophobic group’s interaction with the enzyme. Relatively slow deacylation of β-lactams indicates that they are poor substrates for GlpG, forming a nonproductive structure and explains why a stable acyl enzyme complex could be observed in the crystals.

### Structural Changes in GlpG upon β-Lactam Binding

**Figure 3:**
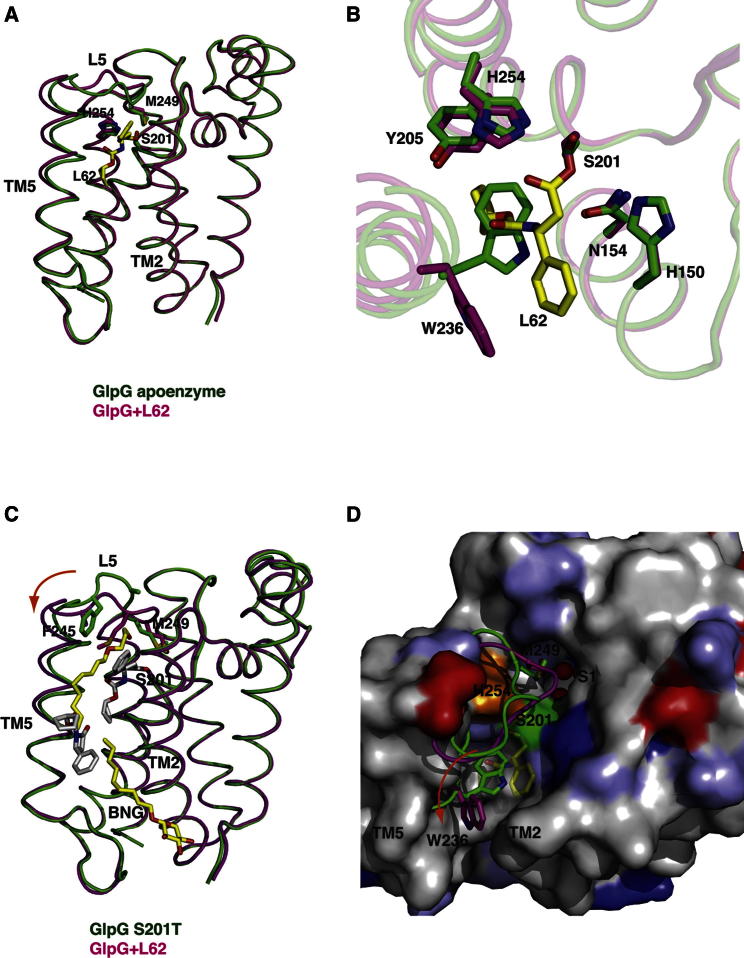
Comparison of GlpG Structures (A) An overlay of GlpG apoenzyme (2XOV) and the β-lactam-bound structure (L62). The overall structural change in the enzyme upon inhibitor binding is minor, with modest changes in TM5 and loop5. The carbon atoms of inhibitor molecule (yellow), the side chains of active site residues (S201 and H254), and M249 from loop5 are shown in stick representation. In both structures, the side chain of M249 points into the active site. (B) A close-up view of the active sites of the apoenzyme and GlpG in complex with β-lactam (L62). Key residues in the active site are represented as sticks, and the carbon atoms are either green (apoenzyme) or magenta (L62). The change in the side chain of W236 upon inhibitor binding is clearly evident. (C) An overlay of the GlpG L62 structure and GlpG S201T (2XTU). The inhibitors, detergent molecules, catalytic serine, and side chains of loop5 are shown in stick representation. The carbon atoms of inhibitor molecules in the L62 structure are white, and detergent molecules in the GlpG S201T structure are yellow. The structural change in TM5 is marked with an arrow. (D) An overlay of loop5 and the side chain of W236 from both GlpG S201T and L62 structures on a surface representation of the GlpG-L62 structure viewed from the periplasm. The inhibitor (L62) is shown in yellow stick representation. The direction of movement of the W236 side chain is shown with an arrow. The structural change in TM5 and W236 is essential for the formation of the S2′ cavity, which is not observed in the GlpG S201T structure, although a detergent molecule protrudes into the active site. Protein is color-coded according to the biochemical properties: positively and negatively charged amino acids in blue and red, respectively, polar amino acids in light blue, and the remainder in gray. See also Figure S5.

Comparison of the β-lactam structures with the apoenzyme shows only a modest structural change, particularly in TM5 and loop5 (Figure 3A). In the L61 and L62 structures, loop5 is partly lifted, with the side chain of M249 still pointing into the active site as in the apoenzyme. Despite the formation of an acylated enzyme and the binding of inhibitor, the active site shows very little change and closely resembles that of the apoenzyme (Figure 3B). The side chain of the active site serine S201 adopts a different rotamer and points away from the catalytic histidine, H254.

In contrast, previous structures of GlpG in complex with isocoumarin or fluorophosphonates showed significant changes at the active site (Vinothkumar et al., 2010; Xue et al., 2012; Xue and Ha, 2012). In particular, the catalytic histidine formed a covalent bond with the isocoumarin and in the presence of fluorophosphonates, moved significantly away from the catalytic serine (Figures S5A and S5B), which also results in the side chain of Y205 adopting a different rotamer. Perhaps due to mode of binding and design of the β-lactam inhibitors, such changes are not observed in the present structures. The position of the side chain of W236 side chain is interesting in these structures. It faces inward in the apoenzyme (Figure 3B) and occupies intermediate positions in isocoumarin and diisopropyl fluorophosphonate (DFP) structures (Figure S5) but swings completely out toward the bilayer in the β-lactam structures.

### Formation of S2′ Cavity

A comparison of the L62 structure and a previously described structure of an active site mutant of GlpG (S201T) highlight the changes necessary for the formation of the S2′ cavity. Like in the L62 structure, the GlpG (S201T) structure shows two detergent molecules at the TM2/TM5 interface; one of these detergents protrudes into the active site (Vinothkumar, 2011). The L62 and GlpG (S201T) structures are largely similar including loop5 with the side chain of M249 pointing into the active site (Figure 3C). Although the bulky hydrophobic detergent molecule points into the active site in the GlpG S201T mutant structure, no S2′ cavity forms. Presumably, because the detergent neither has the right chemical group to form the S2′ cavity, nor can form a covalent bond with catalytic serine, the structural change induced is limited to a minor displacement of loop5 without a change in TM5 (Figure 3C). In contrast, the binding of the inhibitor results not only in partial displacement of loop5, but also a change in TM5 and concurrent movement of the side chain of W236 (Figures 3C and 3D), resulting in a fully formed S2′ cavity, allowing the hydrophobic carbamate group of the ligand to bind. These structures therefore show that while it is possible for any hydrophobic group (for example, a detergent molecule) to interact with GlpG, formation of a productive enzyme complex appears to be dependent on the presence of appropriate chemical motifs.

The observed position of the external ligand is possibly dictated by the crystal packing of GlpG, and the partitioning and position of ligand may differ in presence of the membrane. Nevertheless, the L62 structure provides an opportunity to understand how ligand enters the active site and forms a covalent complex. The initial interaction of the ligand through the TM2/TM5 interface is probably followed by a partial displacement of loop5 and perhaps a change in TM5, which allows access to the catalytic serine. The conformation of the side chain of W236 as observed in the apoenzyme could support the ligand, interacting with the N-substituent of the β-lactam, thus positioning the ligand for the nucleophilic attack. We speculate that the W236 side chain rotates to form the S2′ cavity only after the formation of the ester bond. It is possible that the change in TM5 could be larger when a TM substrate binds. However, from biochemical analysis it is evident that only a subset of residues (for instance P2′-P2 subsites) interact around the active site (Strisovsky et al., 2009), and the rest of the interactions of the TM substrate with the enzyme is likely to be with residues in TM2 and TM5 facing the bilayer. Thus the extent of structural change observed in the β-lactam complexes is probably a good illustration of initial ligand/substrate binding at the active site—that is, binding of the substrate at the TM2/TM5 interface followed by formation of the ester bond and S2′ cavity.

### A Model for Deacylation

**Figure 4:**
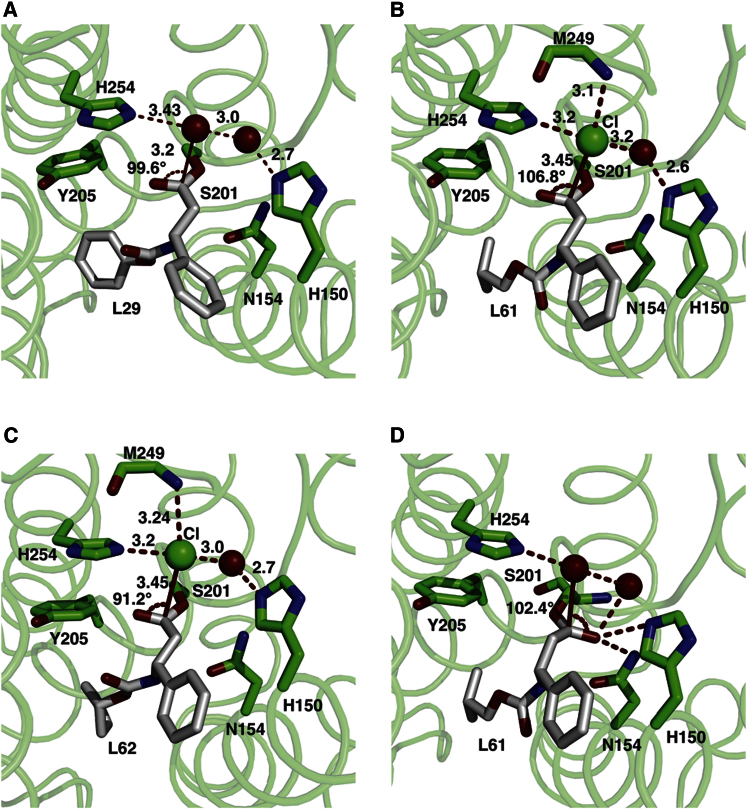
Model for Deacylation (A–C) The interaction of the water/chloride ion with the ligand in the inhibitor structures is shown. The water molecule (red sphere) or chloride ion (green sphere) hydrogen-bond with the side chain of H254, and the water molecule that also hydrogen-bonds to H150. In the L61 and L62 structures, the chloride ion also interacts with main chain amide of M249. Because the carbonyl oxygen points away from the oxyanion hole, they are geometrically unfavorable for deacylation. (D) To model a productive enzyme-inhibitor complex, the carbonyl oxygen in the L61 structure was rotated toward the oxyanion hole so that it is stabilized by hydrogen bonds from H150, N154, and a water molecule. By replacing the chloride ion with a water molecule (red sphere), a structure favorable for deacylation can be generated. See also Figure S6.

In all structures described here, a water molecule (in the L29 structures) or chloride ion (in the L61 and L62 structures) has been modeled close to the covalently bound inhibitor (Figures 4A–4C). Both water and chloride ions are coordinated by hydrogen bonds to the catalytic histidine and water molecule and in the L61 and L62 structures, the main chain amide of M249. In data set1 of the L29 structure, this water molecule has a lower occupancy judged from the density and higher B factor (Figure S6). The observed angle of water/chloride to the carbonyl oxygen of the inhibitor ranges between 91.2° and 106.8° in all the structures described here.

The final step in the proteolytic cycle of serine proteases involves a deacylation step mediated by a water molecule, activated by the catalytic histidine. Because the water/chloride ion hydrogen bond to catalytic histidine and the observed angles are very close to those described by Bürgi and colleagues for the nucleophilic attack of the water on the ester bond (Bürgi et al., 1973), perhaps this indicates the likely position of the water molecule in GlpG for the deacylation step. However because the carbonyl oxygen of the inhibitors faces away from the oxyanion hole, they are geometrically unfavorable for deacylation in the present structures. To model a productive enzyme inhibitor structure, the carbonyl oxygen of the L61 inhibitor was rotated toward the oxyanion hole so that carbonyl oxygen is stabilized by hydrogen bonds from residues H150, N154, and a water molecule. In this geometry, a water molecule placed in the same position as the chloride ion now achieves a favorable conformation for the attack on the ester bond to carry out subsequent deacylation (Figure 4D).

## Discussion

The original discovery of β-lactams as inhibitors of serine proteases was serendipitous; inhibitors originally designed for β-lactamase were also found to inhibit elastase (Doherty et al., 1986; Powers et al., 2002). β-lactam inhibition of GlpG follows a similar mechanism to the inhibition of elastase (Navia et al., 1987; Taylor et al., 1999; Wilmouth et al., 1998) and signal peptidases (Paetzel et al., 1998), where the nucleophilic attack of serine on the carbonyl oxygen at position 2 (Figure 1A) results in ring opening and the formation of an acyl enzyme intermediate. The efficient inhibition of rhomboid proteases by β-lactams is illustrated by the numerous interactions formed between enzyme and inhibitor (Figure 1D; Figure S1), and by the slow deacylation of the acyl enzyme (Figure S4).

SARs of the β-lactams revealed that the chemical substituent at positions 1, 3, and 4 of the β-lactam ring influenced inhibitory activity against rhomboids (Pierrat et al., 2011). With the present structural data, it is now possible to appreciate how this is achieved, as summarized below.

The nature of the chemical group attached to the carbamate of the β-lactams exerts the biggest effect on rhomboid proteases. In the ring-opened form of the β-lactams, the N-substituent at position 1 points into the S2′ cavity and forms extensive interactions with the enzyme, thus explaining why this has a large effect (Figure 2A). The efficiency of inhibition of GlpG increases with increasing hydrophobicity of the N-substituent (Figure 2D); this correlates well with the previous mutagenesis study of rhomboid substrates, which revealed preference for hydrophobic groups at the P2′ position (Strisovsky et al., 2009).

It was also observed that the efficiency of β-lactam inhibition between different rhomboids varied. Some were more potent inhibitors of GlpG, while others were more active against AarA, a rhomboid protease from *Providencia stuartii* (Pierrat et al., 2011). A structure-based alignment of rhomboid proteases reveals differences in the nature of the amino acids that line the S2′ cavity, which would be expected to affect the β-lactam-enzyme interaction and therefore the inhibition (Figure 2A). For example, in AarA the residues that form the wall and the base of the S2′ cavity are replaced with amino acids with smaller side chains thus making the cavity bigger than in GlpG (Figure S7). An analysis of the related GlpG structure from *Haemophilus influenzae* (Figure S8) shows a hydrophobic cavity at the same position as in *E. coli* GlpG (Lemieux et al., 2007), thus a β-lactam with a small hydrophobic group can fit easily into it. Residues from TM2 and TM5 are the least conserved in rhomboids and because they also form part of the S2′ cavity, this local difference could contribute to substrate specificity.

Because the phenyl ring at position 4 of the β-lactam ring points toward the bilayer (Figure 1C), it is not surprising that this substituent was found to be nonessential for activity in the SAR study (Pierrat et al., 2011).

A large extension at position 3 of the β-lactams was found to be detrimental to GlpG inhibition. The orientation of the inhibitor in these structures indicates that this extension would point up toward the solvent, so it is unclear why it should have an effect (Figure 1D). It is possible that a large hydrophobic group at this position may discourage a facile reaction with the enzyme in the initial step.

A comparison of the three distinct classes of inhibitor-bound structures of GlpG reveals their mode of binding at the active site as well as the formation and size of S2′ cavity. The structural change in TM5 is comparable in the isocoumarin and the β-lactam structures (Figure S9). The S2′ cavity in the isocoumarin structure is smaller than those observed with β-lactams, largely due to the position of W236 side chain and the small methoxy substituent (Vinothkumar et al., 2010). The structure of GlpG in complex with DFP shows the smallest change in TM5 among the inhibitor structures, shows no S2′ cavity, and the side chain of W236 occupies a similar position to the apoenzyme but adopts a different rotamer orientation (Figures S10A and S10B) (Xue and Ha, 2012). When a fluorophosphonate with a larger carboxybenzyl (Cbz) group is bound to GlpG, the S2′ cavity is observed and the inhibitor carbonyl oxygen points into it (Figures S10C and S10D) (Xue et al., 2012). These structures further support the proposal that the nature of the chemical groups (size and hydrophobicity) determine the size of the S2′ cavity, defined by changes in TM5 and W236 and accompanied by a partial or complete displacement of loop5.

Chymotrypsin, a digestive serine protease, was included as a selectivity control in the initial screen for rhomboid inhibitors. Some β-lactams were selective against rhomboids while not affecting chymotrypsin (Pierrat et al., 2011). It is notable that substrates digested by chymotrypsin typically have an aromatic group at the P1 residue (Henderson, 1970). One would therefore expect that the hydrophobic groups, either at position 4 or those attached to the carbamate of the β-lactams, are most likely candidates to interact with the S1 cavity of chymotrypsin. Because rhomboids also prefer hydrophobic residues at the P2′ position of substrate, how is the selectivity over chymotrypsin achieved? We propose that the substituent at position 4 of the β-lactams interacts with the S1 cavity of chymotrypsin. This is based on the fact that deletion of the aryl ring at position 4 removes inhibition of chymotrypsin (Pierrat et al., 2011). In contrast, the substituent at position 4 has very little effect on GlpG or AarA. By increasing the length of the hydrophobic linker at position 4, it may be possible to further increase the selectivity for rhomboids. Thus the present structures of GlpG in complex with β-lactams provide a platform for structure-based design of more specific and potent inhibitors for rhomboid proteases.

## Experimental Procedures

Procedures for protein purification, crystallization, and activity assay are described in Vinothkumar et al., 2010 and Pierrat et al., 2011. A complete description of methods can be found in the Supplemental Experimental Procedures.

## Accession Numbers

The coordinates and structure factors have been deposited in the Protein Data Bank with accession numbers 3ZMI (L29 data set 1), 3ZOT (L29 data set 2), 3ZMJ (L61), and 3ZMH (L62).

## Supplemental Information

Document S1. Figures S1–S10, Table S1, and Supplemental Experimental Procedures

3D Molecular Model S1. 3ZMI: Structure of GlpG in Complex with L29, Related to Figure 1B

3D Molecular Model S2. 3ZOT: Structure of GlpG in Complex with L29 (data set2), Related to Figure 4A

3D Molecular Model S3. 3ZMJ: Structure of GlpG in Complex with L61, Related to Figures 1B and 2

3D Molecular Model S4. 3ZMH: Structure of GlpG in Complex with L62, Related to Figures 1B–1E, 3, and 4C
