# Supplementary material for: Structure of Rhomboid Protease in Complex with β-Lactam Inhibitors Defines the S2′ Cavity
Source: Structure. 2013 Jun 4;21(6):1051–8. doi: 10.1016/j.str.2013.03.013 (PMC3690538; doi:10.1016/j.str.2013.03.013)
Supplement: Document S1. Figures S1–S10, Table S1, and Supplemental Experimental Procedures [file mmc1.pdf]

Structure, Volume *21*

Supplemental Information

Structure of Rhomboid Protease in Complex with β-Lactam Inhibitors Defines the S2′ Cavity

Kutti R. Vinothkumar, Olivier A. Pierrat, Jonathan M. Large, and Matthew Freeman

<u>**Inventory of Supplemental Information**</u>

Figure S1: related to Figure 1
Figure S2: related to Figure 1E
Figure S3: related to Figure 2
Figure S4: related to text under sub heading S2' cavity
Figure S5: related to Figure 3
Figure S6: related to Figure 4
Figure S7: related to Discussion
Figure S8: related to Discussion
Figure S9: related to Discussion
Figure S10: related to Discussion
Table S1: related to main text under sub heading structure of GlpG in complex with β-lactams.
Supplemental Experimental Procedures: A complete description of experimental procedures.

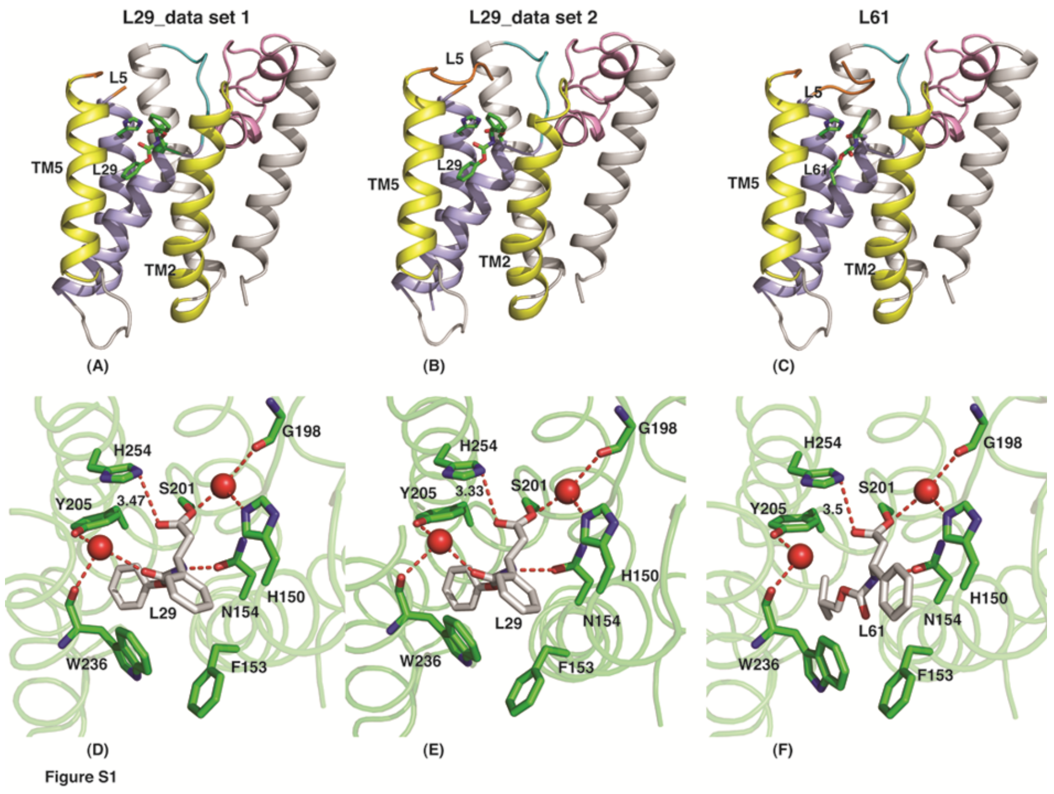

Figure S1: The overall structures of GlpG in complex with β-lactams L29 (A – data set 1 and B data set 2) and L61 (C) (related to Figure 1)

The phenyl group at position 4 points towards the gap between TM2 and TM5 (coloured yellow). The substituent at the carbamate points into the enzyme. The active site residues S201 and H254 and inhibitor molecules are shown in stick representation. All structures described here are similar (Table S1), differences largely localised in the loop regions. Loop5 in the L29 dataset 1 structure is disordered but the main chain residues for F245-M247 could be modelled in the L29 dataset 2. In the L61 structure, except for residue F245 the remaining residues of loop5 (246-249) could be modelled. In the L29 structures, the aromatic ring at position 4 adapts a different orientation than in the L61 and L62 structures.

D, E and F: Interactions of L29 and L61 with GlpG respectively.

Water molecules are shown as red spheres, inhibitor molecules in grey stick representation and the hydrogen bonds are shown as red lines. In the L29 structures (D and E), a water molecule hydrogen bonds to the carbamate oxygen of the inhibitor. In the L61 structure, the carbamate oxygen does not hydrogen bond to any residue or water molecule.

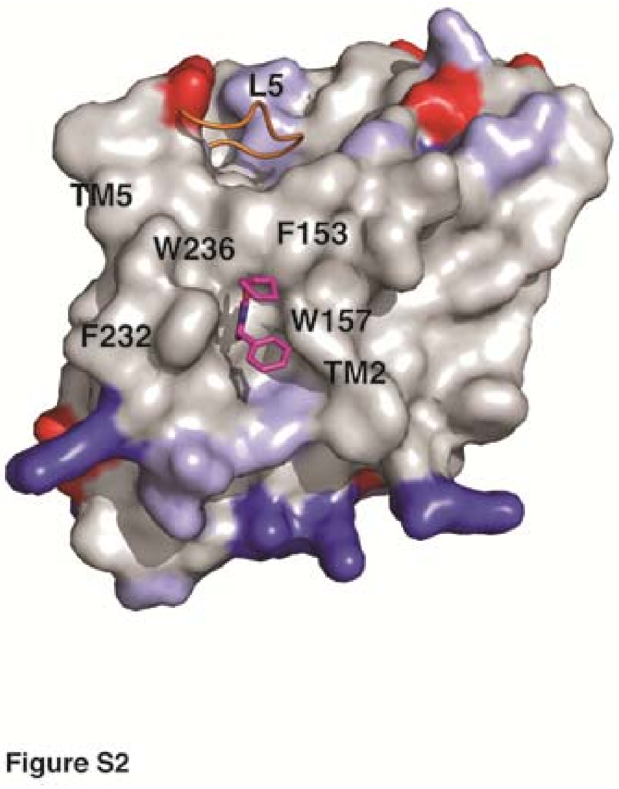

Figure S2: Surface representation showing the uncleaved inhibitor fitting into the groove between TM2/TM5 (related to Figure 1E)

Protein is colour coded according to the biochemical properties: positively and negatively charged amino acids in blue and red respectively, polar amino acids in light blue and the remainder in grey. Key residues that interact with the inhibitor at TM2/TM5 interface are labelled.

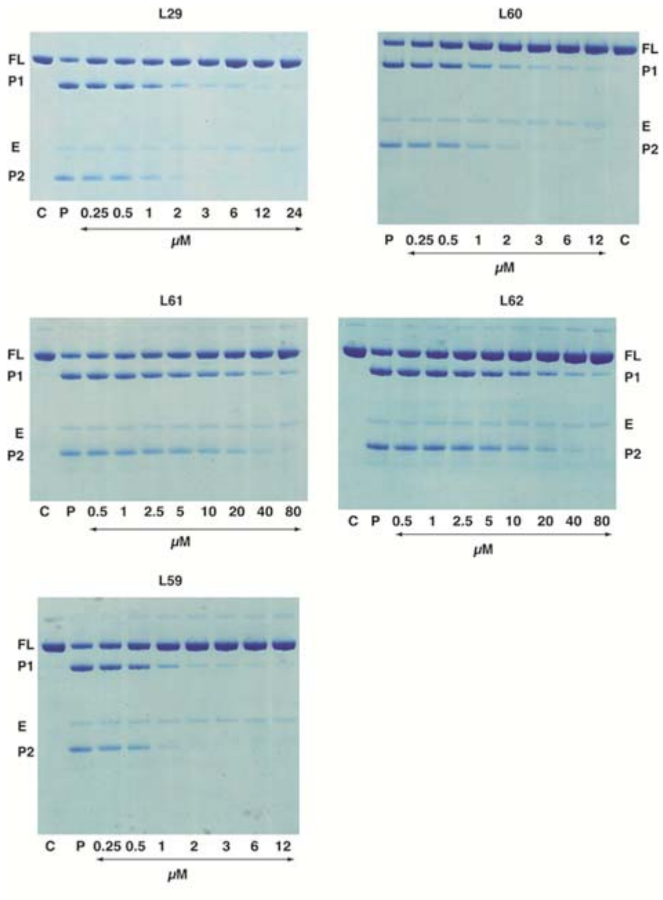

Figure S3

Figure S3: Examples of gels showing β-lactam inhibition of GlpG (related to Figure 2)

Bands from such gels were quantified, and substrate conversion with respect to control was determined. These data were then used to determine the IC_{50} values. Lanes labelled C and P denotes controls with substrate alone or substrate plus enzyme, without inhibitor respectively. In the figures substrate with MBP and thioredoxin fusion (FL), when cleaved by GlpG (E), results in two products (P1 and P2).

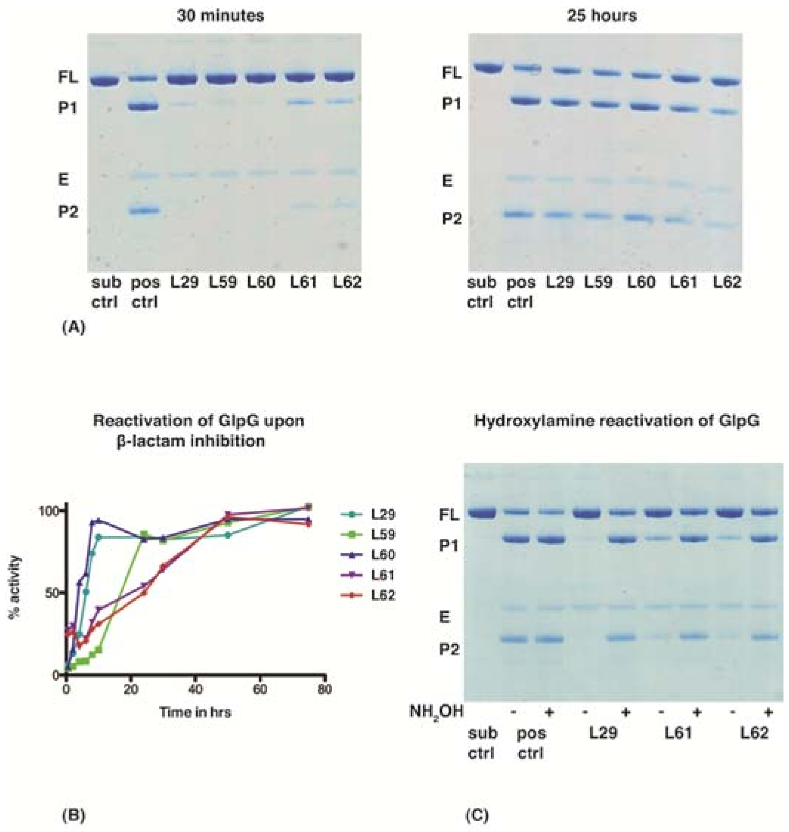

Figure S4

Figure S4: Reactivation of GlpG upon β-lactam inhibition (related to section S2' cavity)

A) GlpG was incubated with an excess of inhibitors for a time period between 30 minutes to 72 hours. Enzyme-inhibitor complex was withdrawn at regular intervals and diluted into a substrate-containing buffer; the recovery of the activity was monitored as a function of time. The reaction mixtures at two time points (30 minutes and 25 hours) are shown as examples. In figures (A and C) substrate with MBP and thioredoxin fusion (FL), when cleaved by GlpG (E), results in two products (P1 and P2).

B) A plot of activity against time shows that L29 and L60 reactivate fastest, 50% of WT activity being recovered in 6 or 3.5 hours respectively. In contrast, inhibitors L61 and L62 with smaller hydrophobic substituents showed slower recovery, 50% of WT activity regained after 24 hours. Inhibitor L59 had an intermediate behaviour, 50% of

WT activity regained after ~17 hours. The slow deacylation from GlpG show that β-lactams are poor substrates.

C) The recovery of GlpG activity is accelerated by incubation with hydroxylamine. When enzyme-inhibitor was incubated with 0.4 M hydroxylamine for 30 minutes, 70-80% activity was recovered. In contrast, incubation with buffer alone for the same period showed no recovery of the activity. Lanes labelled as pos ctrl denotes enzyme-substrate mixture with no inhibitor added.

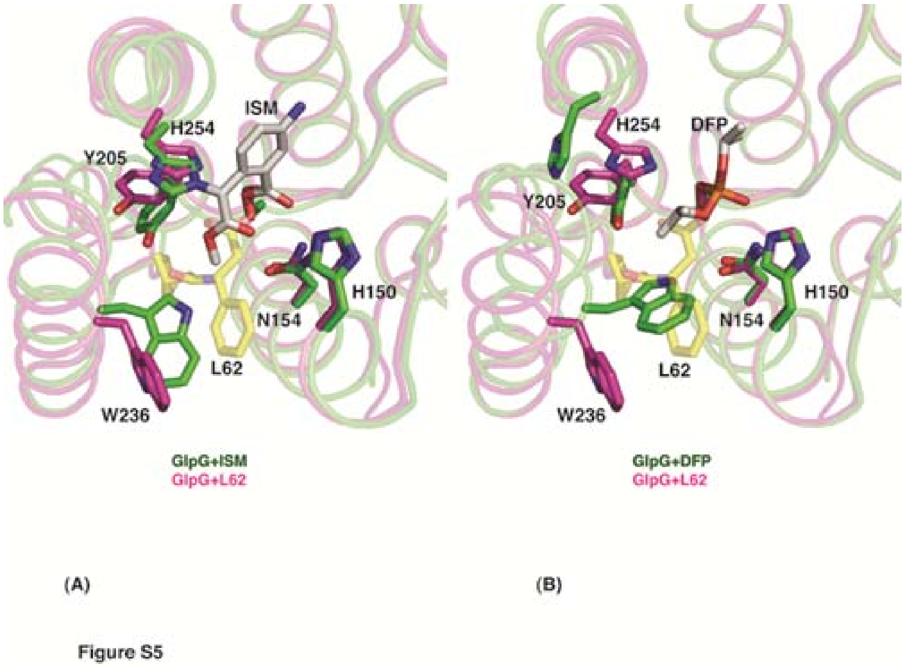

Figure S5: Comparison of the active site of GlpG bound with different inhibitors (related to Figure 3)

The active sites of GlpG with doubly bonded isocoumarin inhibitor (2XOW) and diisopropyl fluorophosphonate (DFP) (3TXT) superimposed on the L62 structure are shown in panels A and B respectively. The carbon atoms of side chains and inhibitor molecules in isocoumarin (ISM) and DFP are coloured in white. The displacement of H254, either due to the formation of covalent bond with isocoumarin or the presence of DFP, and the associated rotation of Y205, are absent in the L62 and other β-lactam structures. Note that in the isocoumarin and fluorophosphonates structures, the oxygen atoms of the inhibitors point towards the oxyanion hole and thus the water molecule hydrogen bonded to H150 in β-lactam structures (Figure 1D and Figure S1) is displaced (Vinothkumar et al., 2010; Xue et al., 2012; Xue and Ha, 2011). The side chain of W236 occupies an intermediate conformation in the isocoumarin structure and adapts a different rotamer in DFP structure. In these conformations, the side chain of W236 would obstruct the binding of the hydrophobic substituent of β-lactams. Unlike isocoumarins and fluorophosphonates, β-lactams do not possess a chemical motif that interacts upstream of the catalytic serine, so the structural change is confined to TM5 and W236. This is also supported by the conformation of loop5 where the side chain of M249 still points towards the active site and plugs the S1 cavity (Figure 3A).

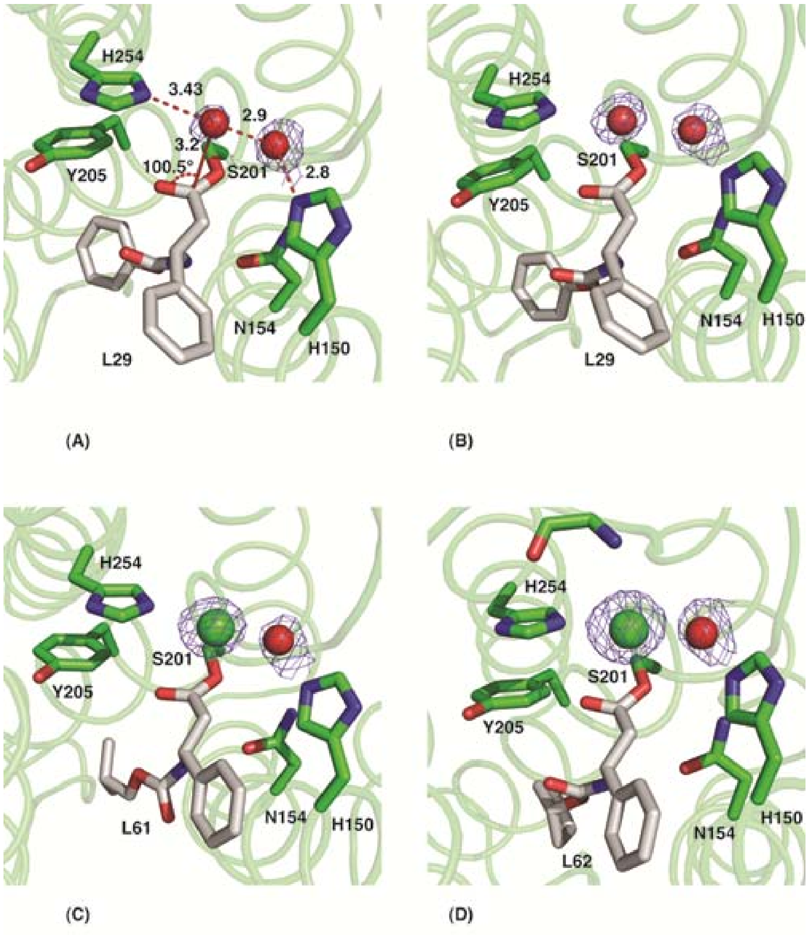

Figure S6

Figure S6: Electron density maps of water /chloride molecules close to the β-lactam inhibitors (related to Figure 4)

Panels A-D show 2Fo-Fc maps (1σ) drawn around water/chloride molecules at the active site and close to the β-lactam inhibitors. For comparison, the density of water molecule hydrogen bonded to H150 is also shown. Panels A and B show water molecules modelled in the two L29 structures. In data set 1 of the inhibitor L29 (A), the water molecule close to the C2 atom of the inhibitor L29 has a partial occupancy reflected also in the B factor (54.48 $Å^2$), while an adjacent water molecule hydrogen bonded to H150 refines to a B factor of 36.61 $Å^2$. In data set 2 of the inhibitor L29 (B), the water molecule close to C2 atom of the inhibitor L29 is better ordered (B factor - 33.9 $Å^2$) and the water hydrogen bonded to H150 refines to a B factor of 26.5 $Å^2$. Panels C and D shows the density around chloride ion and water in the L61 and L62 structures.

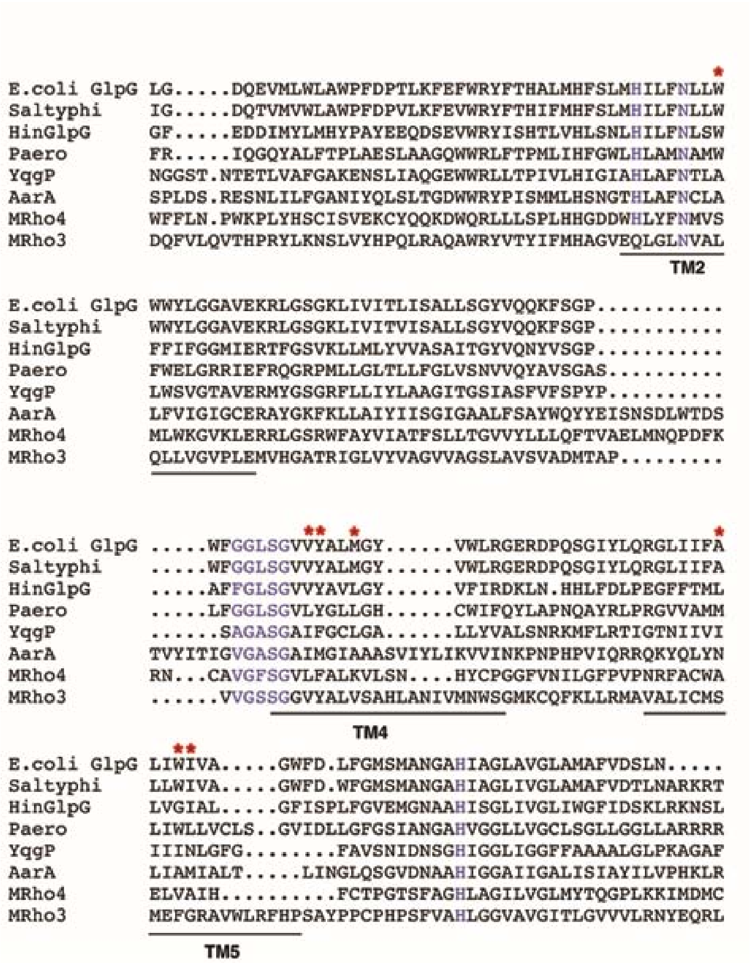

Figure S7

Figure S7: Structure based alignment of rhomboid proteases (related to discussion)

The coordinate of *E.coli* GlpG (2XOV) was used as a template for alignment. Prokaryotic rhomboids from *Salmonella typhi* (Saltyphi), *Haemophilus influenzae* (HinGlpG), *Pseudomonas aeruginosa* (Paero), *Bacillus subtilis* (YqgP) and *Providencia stuartii* (AarA) and two mouse rhomboids (MRho3 and MRho4) have been included. The TM region of helices 2, 4 and 5 are underlined. The conserved residues H150 and N154 in TM2, GxSG motif in TM4 and H254 are coloured in blue (the numbers correspond to *E.coli* GlpG). The amino acids that form the wall (W157, Y205, W236), the base (M208) and the residues whose side chains (V204, A232, I237) point into and form part of S2' cavity are marked with red asterisks. It should be noted that TM5 is flexible and the conformation of TM5 in rhomboids could also dictate the nature of the S2' cavity. The alignment was made using STRAP.

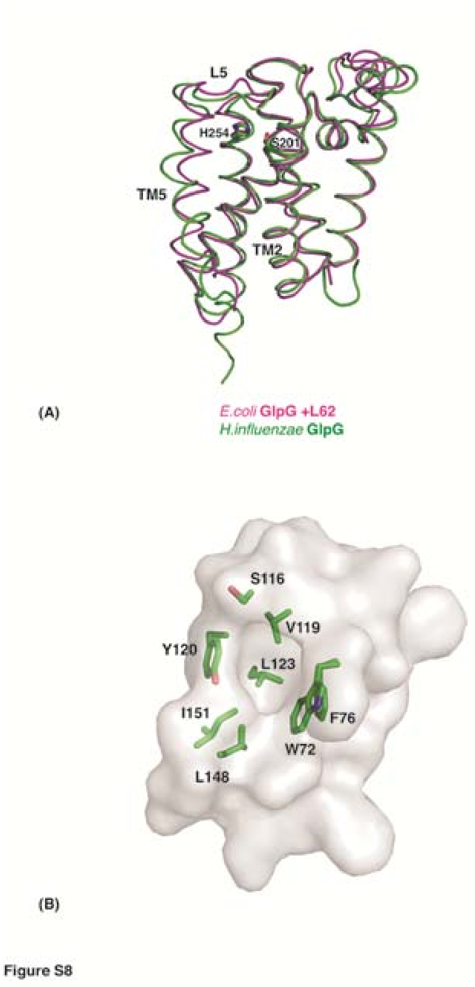

Figure S8: Comparison of *E.coli* GlpG in complex with L62 and *Haemophilus influenzae* GlpG (related to discussion)

A) Overlay of *H.influenzae* GlpG (2NR9) and *E.coli* GlpG L62 structure. Although, there is no inhibitor or substrate bound to *H.influenzae* GlpG, TM5 adopts a more open structure than the inhibitor bound structure of *E.coli* GlpG. (B) Perhaps due to this open structure, a cavity is observed in the same postion as in *E.coli* GlpG. The base of the S2' cavity in *H.influenzae* GlpG is formed by L132. The wall of the cavity is formed by Y120, W72 and F76. The side chains of residues L148 and I151 in TM5 also point into the cavity as in *E.coli* GlpG.

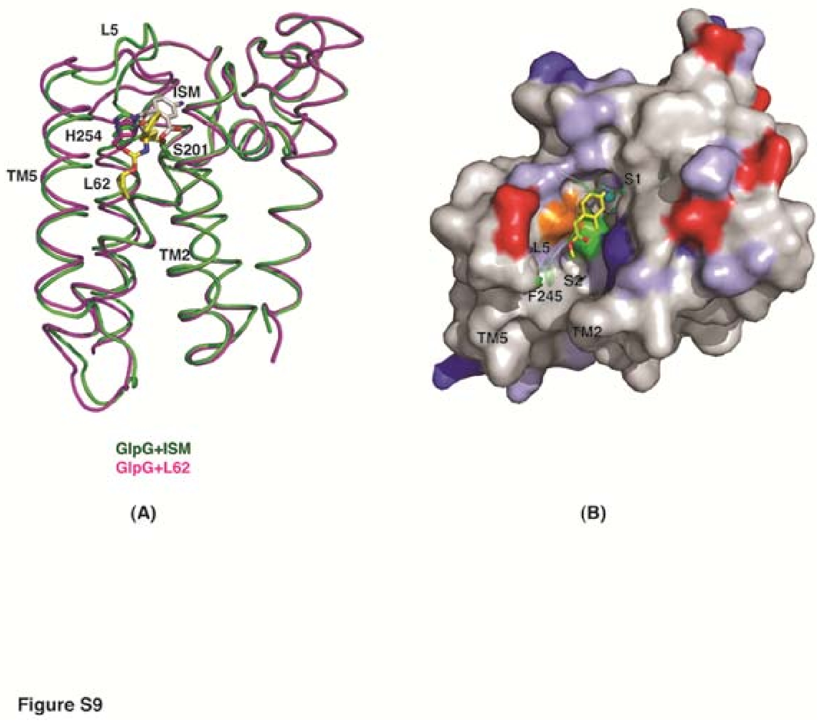

Figure S9: Comparison of GlpG in complex with L62 and isocoumarin (related to discussion)

A) Overlay of β-lactam and isocoumarin structures (2XOW). The structural change in TM5 is comparable, while loop5 is more open in the isocoumarin structure.

B) Surface representation of isocoumarin bound GlpG structure showing the S2' cavity (colour coded as in figure S2). Loop5 shown in transparent green covers the inhibitor.

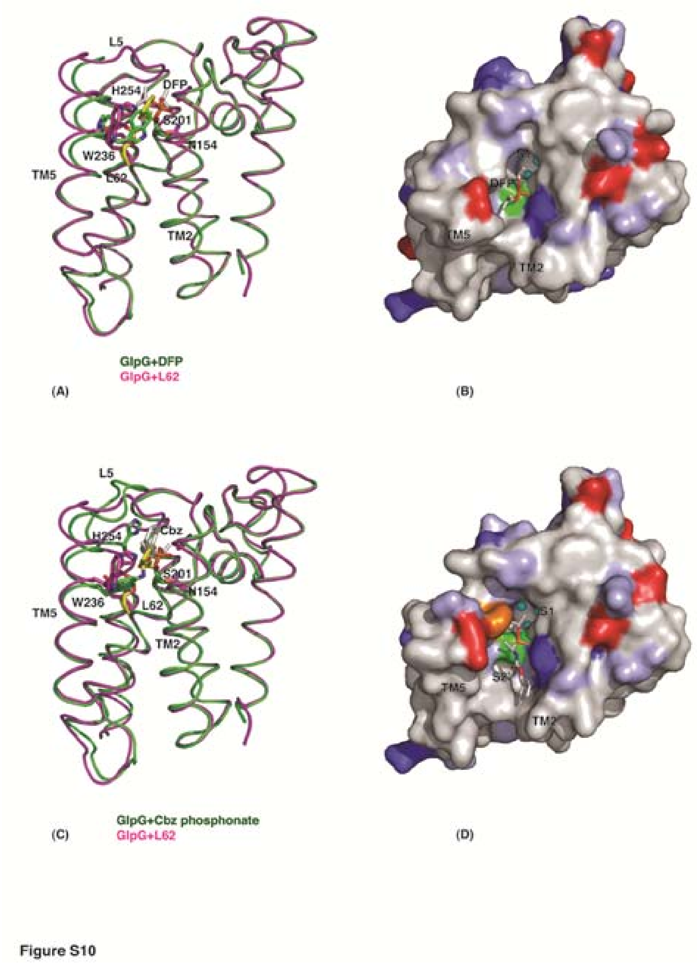

Figure S10: Comparison of GlpG in complex with L62 and fluorophosphonates (related to discussion)

A) and C) Overlay of β-lactam and fluorophosphonate structures (PDB codes for DFP and Cbz-fluorophosphonates are 3TXT and 3UBB respectively). Part of loop5 in the DFP structure is disordered, and the change in TM5 is minimal, when compared to the apoenzyme. The Cbz-fluorophosphonate structure shows a change in TM5 similar to β-lactam. The Cα position of W236 is very similar, however the side chain in this structure still obstructs and would prevent binding of the β-lactam. The inhibitor molecules and key amino residue side chains are shown in stick representation. The carbon atoms of DFP and Cbz-fluorophosphonate are shown in white and L62 in yellow.

B and D) Surface representations of GlpG in complex with DFP and Cbz-fluorophosphonate structures viewed from the periplasm (colour coded as in figure S2). In the DFP structure, the S2' cavity is not formed. In the Cbz-fluorophosphonate

structure the carbonyl oxygen of the inhibitor points into this cavity, which is small in comparison to the β-lactam structures.

Supplementary table 1: (related to main text – section structure of GlpG in complex with β-lactams)

Reference model used for comparison is the apoenzyme structure of GlpG (2XOV). The root mean square deviation (rmsd) for all the Cα atoms (including residues from loop5) was used in the calculations.

Model	RMSD (Å)
L29 (data set 1)	0.336
L29 (data set 2)	0.47
L61	0.44
L62	0.47

Comparison of GlpG structures in complex with β-lactams:

Model	RMSD (Å)
L29 (data set 1)- L29 (data set 2)	0.15
L29 (data set 1)-L61	0.21
L29 (data set 1)-L62	0.26
L29 (data set 2)-L61	0.2
L29 (data set 2)-L62	0.25
L61-L62	0.14

Materials and Methods

Proteins and inhibitors:

Expression and purification of GlpG and LacY TMD (MBP-TMD-Trx-His6) fusion was carried out as described previously (Pierrat et al., 2011; Strisovsky et al., 2009; Vinothkumar et al., 2010). β-lactams were prepared by treating commercially available 4-phenyl-2-azetidinone with the appropriate chloroformate under basic conditions as described in Pierrat 2011.

β-lactam inhibition and reactivation assay:

A typical rhomboid inhibition assay was performed with full length GlpG (350 nM) and substrate (3.5 μM) in a 10μl reaction buffer containing 25 mM Hepes buffer pH7.5, 0.4 M NaCl, 5% glycerol, 5 mM EDTA, 5% DMSO and 0.05% DDM. Freshly diluted inhibitors were incubated with enzyme for 30 minutes at room temperature (RT). The substrate-enzyme-inhibitor mixture was incubated at 37°C for 45 minutes and the cleavage products were separated on a 10% bis-tris gel (Invitrogen) developed with MES buffer and bands stained with Coomassie brilliant blue. Gels were scanned and bands quantified with ImageJ and analysed with Graphpad Prism version 5.0. The IC_{50} values were determined by fitting the data to a 4-parameter logistic equation.

For the reactivation assay, 100 μM of freshly diluted inhibitor was mixed with enzyme and incubated at RT. The enzyme-inhibitor mixture was withdrawn at regular time intervals and diluted into 20 μl of buffer (the final concentrations of enzyme, substrate and inhibitors were 320 nM, 1.3 μM and 5 μM respectively). The cleavage products were analysed as described above and percentage activity recovered was plotted against time. For the hydroxylamine reactivation assay, the enzyme-inhibitor mixture (100 μM inhibitor and 3.2 μM enzyme in a 25 μl volume) was incubated for 30 minutes at RT, followed by either addition of 0.4 M buffered hydroxylamine (from a freshly made stock of 2M) or an equivalent amount of buffer. This mixture was further incubated for 30 minutes at RT. The enzyme-inhibitor mixture complex (treated, with or without hydroxylamine), was then diluted into a substrate-containing buffer (final concentrations of enzyme, substrate and inhibitors are the same as in the reactivation assay), incubated at 37 °C for 45 minutes and cleavage products separated on gels.

Crystallisation:

Crystals of truncated GlpG WT in β-D-nonyl glucoside were obtained by mixing a solution of 1.5-3M ammonium chloride or 1.5-2M sodium chloride in 0.1 M Bis-Tris, pH7.0 with protein at ratio of 1:1 in hanging drops at 25 °C (Vinothkumar et al., 2010; Wang et al., 2007). Inhibitors were dissolved in 100% DMSO at a concentration of 20 mM and stored at -20 °C. Inhibitors from this stock were diluted to 1.5 mM in buffer resembling the mother liquor with 10% or 20% DMSO just before soaking. The best diffracting crystals and structures were determined from soaking crystals with inhibitor concentrations of 0.8 mM, 0.75 mM and 1.5 mM for L29, L61 and L62 respectively for 30 minutes at RT. All crystals were cryo-protected by adding 25% glycerol to the mother liquor and flash frozen in liquid nitrogen.

Crystallography:

Data sets were collected at the I02/I24 beam line at the Diamond Light Source (Harwell). Diffraction data were indexed and integrated with imosflm and reduced with SCALA or Aimless (Battye et al., 2011; Evans, 2006; Evans, 2011). All four data sets were isomorphous to the apoeznyme data set hence freeRflag was imported from the apoenzyme data (PDB -2XOV) and used as test set during refinement. The coordinate of GlpG (2XOV) with loop5, corresponding to residues 245-249 omitted was used as an initial input model for Phaser (McCoy et al., 2007). Restrained refinement was carried with PHENIX (Adams et al., 2010) and REFMAC5 (Murshudov et al., 2011), followed by manual model building in COOT (Emsley and Cowtan, 2004). Model and library files of the inhibitors were generated with Phenix elbow (Moriarty et al., 2009) or sketcher in CCP4 suite (1994) and link files were generated with Jligand (Lebedev et al., 2012). All figures were made with Pymol (Delano, 2002). Structure based alignment of rhomboid proteases was performed with STRAP (Gille and Frommel, 2001).

All compounds used in soaking are racemic mixtures. When modelling the ligand into the density both enantiomers were considered but the best fit for the density was observed for the R-enantiomer. It is possible for the S-enantiomer to bind without any clashes, which will probably result in the carbamate oxygen hydrogen bonding to N154 and the nitrogen atom of the inhibitors facing away.

Refinement of the L29 structures: In data set 1 of the L29 inhibitor, the water molecule modelled close to the C2 atom of the L29 inhibitor was not picked automatically by refinement program but the presence of positive density (~2.7 σ in Fo-Fc maps generated with Phenix and also cross-checked with refinement with Refmac) at this position prompted us to model water molecule. The density of this water molecule is poor, indicating a partial occupancy. To verify, if the density is genuine and not noise, we refined two independent data sets of crystals soaked with L29. These data sets show a strong density for the water molecule and one of these data sets has been deposited (3ZOT) and called L29 data set 2. The distance of the water molecule and the C2 atom of the inhibitor in both data sets were restrained to 3.2 Å (without this restraint the distance between the water molecule and the C2 atom of the inhibitor is between 2.8-3 Å). Additionally, in the L29 structures a density is observed over the active site and around the S1 cavity and could represent the side chain of M249. However, as the density for the residues 245-249 corresponding to loop5 are poor this has not been modelled. The final model of the L29 structures do not contain any ramachandran outliers and have a clash score of 1.73 (data set 1) and 1.97 (data set 2).

Refinement of the L61 structure: In the L61structure, the spherical density observed close to the C2 atom of the inhibitor was initially modelled as a water molecule (Figure 4B). But the presence of positive density after refinement or indication in COOT as unmodelled blob prompted us to model chloride ion instead due to its abundance in crystallisation and freezing solutions. The distance between the chloride ion and the C2 atom of the inhibitor was restrained to 3.45 Å during refinement. The final model of the L61 structure does not contain any ramachandran outliers and has a clash score of 1.67

Refinement of the L62 structure: Similar to the L61 structure, the spherical density above the C2 atom of the inhibitor was modelled as chloride ion and distance restraint to 3.45 Å (Figure 4C). For the density of the externally bound ligand in L62 structure, both enantiomers were tried but the best fit was observed for the S-enantiomer. Whether both enantiomers of monobactams react with GlpG and if both enantiomers can bind to the active site remains to be verified. The final model of the L62 structure does not contain any ramachandran outliers and has a clash score of 1.99.

Generating a model for deacylation: To generate a model for deacylation, the carbonyl oxygen of the L61 inhibitor was rotated towards the oxyanion hole and refined with the same library and link files of the inhibitor. This results in the model depicted in figure 4D. We also note that H150 and the water molecule hydrogen bonded to this residue could in principle play a role in deacylation. However, the distance between this water molecule and the C2 atom in the present structures is between 3.5-4.1 Å and in the model described in the main text for deacylation (Figure 4D), the angle to C2-O of the inhibitor from this water molecule is not optimal (~55°) for it to act as a nucleophile for the hydrolysis of ester bond (Burgi et al., 1973). Thus we think H150 is unlikely to play a role as the residue activating the water molecule.

Supplementary references:

(1994). The CCP4 suite: programs for protein crystallography. Acta Crystallogr D Biol Crystallogr *50*, 760-763.

Adams, P.D., Afonine, P.V., Bunkoczi, G., Chen, V.B., Davis, I.W., Echols, N., Headd, J.J., Hung, L.W., Kapral, G.J., Grosse-Kunstleve, R.W., *et al.* (2010). PHENIX: a comprehensive Python-based system for macromolecular structure solution. Acta Crystallogr D Biol Crystallogr *66*, 213-221.

Battye, T.G., Kontogiannis, L., Johnson, O., Powell, H.R., and Leslie, A.G. (2011). iMOSFLM: a new graphical interface for diffraction-image processing with MOSFLM. Acta Crystallogr D Biol Crystallogr *67*, 271-281.

Burgi, H.B., Dunitz, J.D., and Shefter, E. (1973). Pharmacological implications of the conformation of the methadone base. Nat New Biol *244*, 186-187.

Delano, W.L. (2002). The PyMOL Molecular Graphics System (http://www.pymol.org).

Emsley, P., and Cowtan, K. (2004). Coot: model-building tools for molecular graphics. Acta Crystallogr D Biol Crystallogr *60*, 2126-2132.

Evans, P. (2006). Scaling and assessment of data quality. Acta Crystallogr D Biol Crystallogr *62*, 72-82.

Evans, P.R. (2011). An introduction to data reduction: space-group determination, scaling and intensity statistics. Acta Crystallogr D Biol Crystallogr *67*, 282-292.

Gille, C., and Frommel, C. (2001). STRAP: editor for STRuctural Alignments of Proteins. Bioinformatics *17*, 377-378.

Lebedev, A.A., Young, P., Isupov, M.N., Moroz, O.V., Vagin, A.A., and Murshudov, G.N. (2012). JLigand: a graphical tool for the CCP4 template-restraint library. Acta Crystallogr D Biol Crystallogr *68*, 431-440.

McCoy, A.J., Grosse-Kunstleve, R.W., Adams, P.D., Winn, M.D., Storoni, L.C., and Read, R.J. (2007). Phaser crystallographic software. J Appl Crystallogr *40*, 658-674.

Moriarty, N.W., Grosse-Kunstleve, R.W., and Adams, P.D. (2009). electronic Ligand Builder and Optimization Workbench (eLBOW): a tool for ligand coordinate and restraint generation. Acta Crystallogr D Biol Crystallogr *65*, 1074-1080.

Murshudov, G.N., Skubak, P., Lebedev, A.A., Pannu, N.S., Steiner, R.A., Nicholls, R.A., Winn, M.D., Long, F., and Vagin, A.A. (2011). REFMAC5 for the refinement of macromolecular crystal structures. Acta Crystallogr D Biol Crystallogr *67*, 355-367.

Pierrat, O.A., Strisovsky, K., Christova, Y., Large, J., Ansell, K., Bouloc, N., Smiljanic, E., and Freeman, M. (2011). Monocyclic beta-lactams are selective, mechanism-based inhibitors of rhomboid intramembrane proteases. ACS Chem Biol *6*, 325-335.

Strisovsky, K., Sharpe, H.J., and Freeman, M. (2009). Sequence-specific intramembrane proteolysis: identification of a recognition motif in rhomboid substrates. Mol Cell *36*, 1048-1059.

Vinothkumar, K.R., Strisovsky, K., Andreeva, A., Christova, Y., Verhelst, S., and Freeman, M. (2010). The structural basis for catalysis and substrate specificity of a rhomboid protease. EMBO J *29*, 3797-3809.

Wang, Y., Maegawa, S., Akiyama, Y., and Ha, Y. (2007). The role of L1 loop in the mechanism of rhomboid intramembrane protease GlpG. J Mol Biol *374*, 1104-1113.

Xue, Y., Chowdhury, S., Liu, X., Akiyama, Y., Ellman, J., and Ha, Y. (2012). Conformational change in rhomboid protease GlpG induced by inhibitor binding to its S' subsites. Biochemistry *51*, 3723-3731.

Xue, Y., and Ha, Y. (2011). Catalytic mechanism of rhomboid protease GlpG probed by 3,4-dichloroisocoumarin and diisopropyl fluorophosphonate. J Biol Chem *287*, 3099-3107.
